# Fallopian tube recanalization: is technical patency enough?

**DOI:** 10.1186/s42155-026-00781-w

**Published:** 2026-10-10

**Authors:** Vinicius Adami Vayego Fornazari, Renato Abu Hana, Grit Armstrong Adler, Sara Lojo-Lendoiro, Gregory Kirk Lewis, Gloria Maria Salazar

**Affiliations:** 1 Division of Vascular and Interventional Radiology, Department of Radiology, College of Medicine, University of Florida, Jacksonville, USA; 2 Complexo hospitalario de Ferrol Interventional Radiologist, Radiology Department, Hospital Arquitecto Marcide, Av. da Residencia, S/N, Ferrol, A Coruña 15405 Spain; 3 Division of Minimally Invasive Gynecologic Surgery, Department of Obstetrics and Gynecology, University of Florida College of Medicine, Jacksonville, FL USA; 4 Division of Vascular Interventional Radiology, Department of Radiology, University of North Carolina, Chapel Hill, NC USA

Dear Editor,

Roberts’ review in *CVIR Endovascular* established fluoroscopy-guided fallopian tube recanalization (FTR) as a minimally invasive option for appropriately selected women with proximal tubal occlusion [1]. It also highlighted a persistent limitation of the literature: technical success is well characterized, whereas pregnancy outcomes are more variable and influenced by heterogeneous populations, competing causes of infertility, and incomplete follow-up. This distinction raises a complementary question: after tubal patency is restored, which patients are actually likely to derive reproductive benefit?

Proximal tubal obstruction is one of the few potentially reversible causes of tubal-factor infertility. FTR can achieve high technical success, yet technical success and reproductive success are fundamentally different endpoints. A systematic review and meta-analysis of 27 observational studies involving 1720 women reported pooled clinical pregnancy and live-birth rates of 27% and 22%, respectively, after tubal catheterization for proximal obstruction [3]. Free intraperitoneal spill confirms restored patency, but does not by itself predict conception.

Patient selection therefore begins before guidewire manipulation. Apparent proximal occlusion on hysterosalpingography (HSG) is not synonymous with real obstruction: cornual spasm, mucus or debris, and preferential contralateral contrast passage may produce proximal non-filling. Selective salpingography should precede recanalization because controlled ostial catheterization may demonstrate patency or relieve functional obstruction [1]. When persistent proximal obstruction is confirmed, the procedural endpoint should be opacification beyond the treated segment with free intraperitoneal spill rather than guidewire passage alone.

The more consequential question is whether restored patency is likely to alter the patient’s reproductive trajectory. Pregnancy after technically successful FTR depends on factors the intervention cannot correct, including age, ovarian reserve, duration and etiology of infertility, endometriosis, distal tubal integrity, uterine disease, and male-factor infertility [2, 3]. Accordingly, these variables should not be interpreted as absolute contraindications to FTR, but rather as factors that may reduce the expected reproductive benefit of restoring tubal patency. Patients with hydrosalpinx or substantial distal tubal disease, diminished ovarian reserve, advanced reproductive age, severe male-factor infertility, or multiple competing causes of infertility should undergo individualized multidisciplinary fertility assessment before FTR is selected as the preferred strategy [3, 5]. Age and ovarian reserve should be incorporated into individualized reproductive assessment; in the absence of validated FTR-specific thresholds, they should not be used as rigid standalone criteria for procedural eligibility. A practical framework is summarized in Fig. 1 and Tables 1 and 2.

**Fig. 1:**
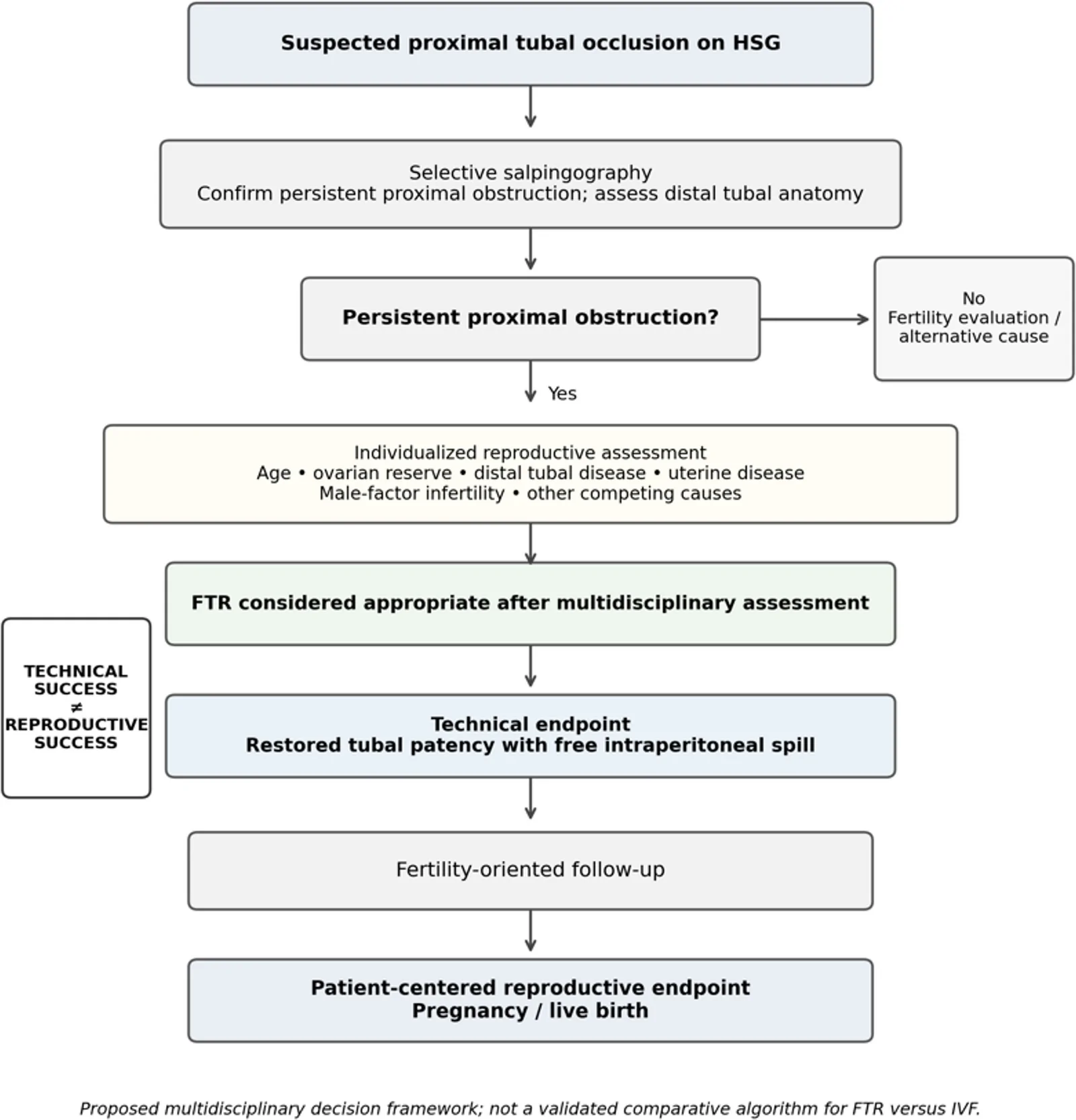
Patient-selection pathway for fallopian tube recanalization (FTR) and expected reproductive benefit. Selective salpingography distinguishes persistent proximal obstruction from functional non-filling and permits assessment of distal tubal anatomy. When fixed proximal obstruction is confirmed, reproductive factors should be assessed before recanalization. Technical success is defined anatomically by restored tubal patency with free intraperitoneal spill; pregnancy and live birth remain distinct, patient-centered reproductive endpoints. The pathway is proposed as a pragmatic multidisciplinary decision framework and is not a validated comparative algorithm for FTR versus IVF

**Table 1:**
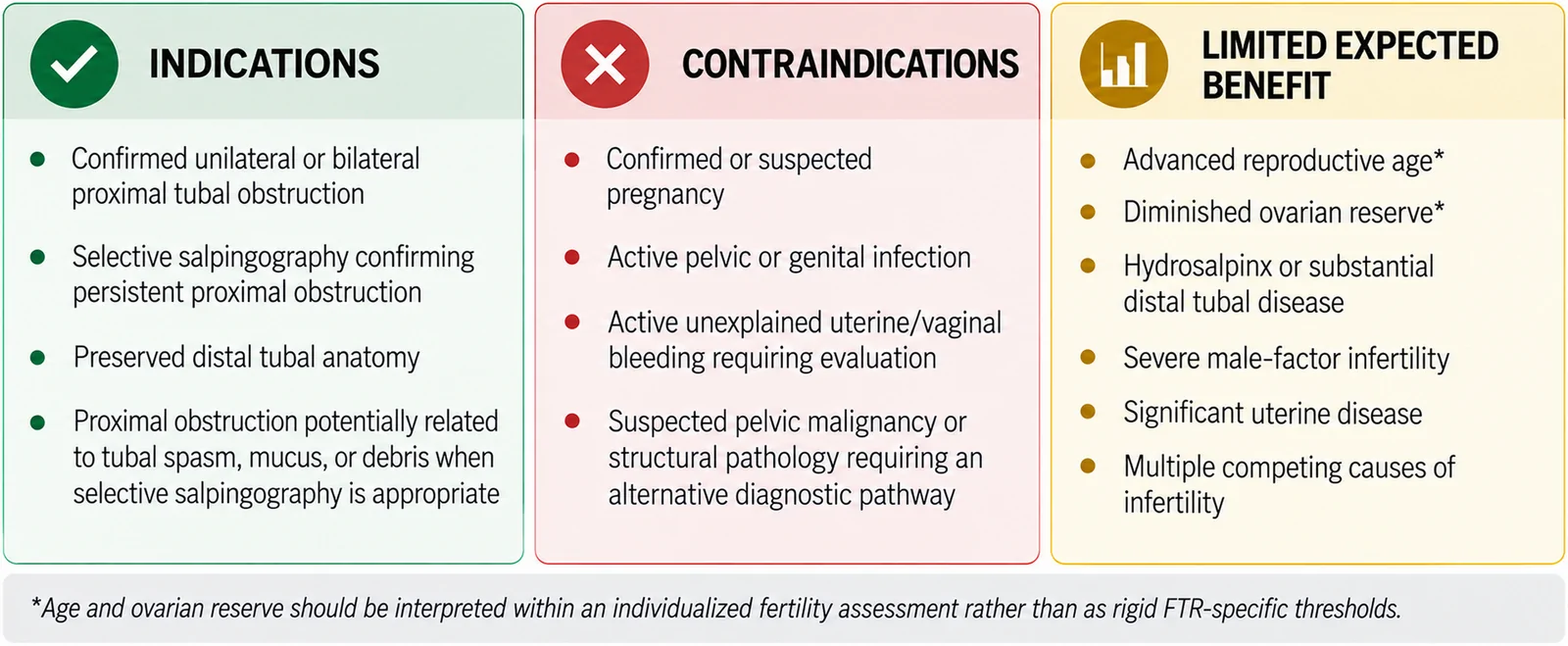
Patient selection and expected reproductive benefit after fallopian tube recanalization. Proposed framework distinguishing appropriate indications for fallopian tube recanalization (FTR), contraindications to intervention, and clinical factors associated with limited expected reproductive benefit despite potential technical feasibility. Factors such as advanced reproductive age and diminished ovarian reserve should be interpreted within an individualized fertility assessment rather than as rigid FTR-specific thresholds. Limited expected benefit does not represent an absolute contraindication to FTR but should prompt multidisciplinary evaluation of competing infertility factors and alternative reproductive strategies. *FTR*, fallopian tube recanalization

**Table 2:**
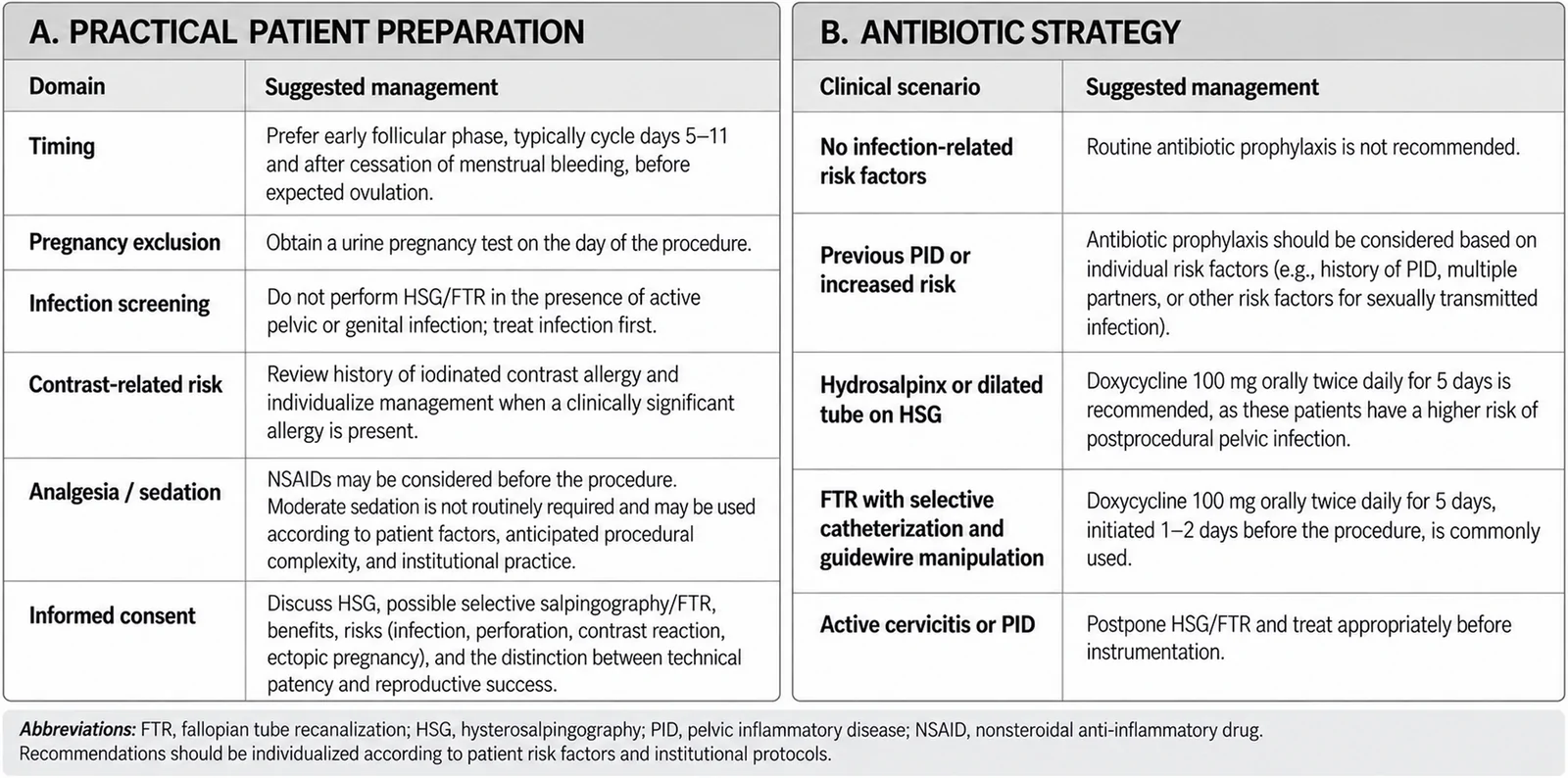
Practical patient preparation and antibiotic strategy for hysterosalpingography and fallopian tube recanalization. (A) Key considerations for patient preparation before hysterosalpingography (HSG) and potential fallopian tube recanalization (FTR), including procedural timing, pregnancy exclusion, infection screening, contrast-related risk, analgesia/sedation, and informed consent. (B) Risk-adapted antibiotic strategy according to infection-related factors and procedural characteristics. Recommendations should be individualized according to patient risk factors and institutional protocols. *FTR*, fallopian tube recanalization; *HSG*, hysterosalpingography; *PID*, pelvic inflammatory disease; *NSAID*, nonsteroidal anti-inflammatory drug

FTR and in vitro fertilization (IVF) should not be framed as competing procedures. FTR attempts to restore native tubal patency and may permit spontaneous conception, whereas IVF bypasses tubal function. Available evidence remains largely observational and heterogeneous; FTR should therefore not be presented as universally equivalent or superior to IVF [3]. Accordingly, the pathway proposed in Fig. 1 should be interpreted as a pragmatic multidisciplinary decision framework rather than a validated comparative algorithm for selecting FTR versus IVF. Its purpose is to integrate anatomical findings with established reproductive factors when considering whether restoration of tubal patency is likely to provide meaningful clinical benefit [4].

Standard preprocedural assessment should include pregnancy exclusion and evaluation for active pelvic infection, with antimicrobial prophylaxis considered according to individual infection risk and institutional practice. After successful recanalization, fertility-oriented follow-up should reassess age, ovarian reserve, semen analysis, ovulation, uterine factors, distal tubal disease, and possible reocclusion when pregnancy does not occur. Recent observational data support clinically meaningful spontaneous conception after successful FTR in selected women [4].

Roberts appropriately emphasized the diagnostic and therapeutic value of selective salpingography and FTR [1]. We propose a complementary perspective: technical success should not be considered synonymous with reproductive success. Although restoration of proximal tubal patency is a well-established procedural endpoint, the more clinically relevant question is whether reopening the tube meaningfully alters the patient’s reproductive trajectory.

## Data Availability

Data sharing is not applicable to this article, as no datasets were generated or analyzed.
